# The role of cation-dependent chloride transporters in neuropathic pain following spinal cord injury

**DOI:** 10.1186/1744-8069-4-36

**Published:** 2008-09-17

**Authors:** Samuel W Cramer, Christopher Baggott, John Cain, Jessica Tilghman, Bradley Allcock, Gurwattan Miranpuri, Sharad Rajpal, Dandan Sun, Daniel Resnick

**Affiliations:** 1 Department of Neurosurgery, University of Wisconsin School of Medicine and Public Health, Madison, WI 53792, USA

## Abstract

**Background:**

Altered Cl^- ^homeostasis and GABAergic function are associated with nociceptive input hypersensitivity. This study investigated the role of two major intracellular Cl^- ^regulatory proteins, Na^+^-K^+^-Cl^- ^cotransporter 1 (NKCC1) and K^+^-Cl^- ^cotransporter 2 (KCC2), in neuropathic pain following spinal cord injury (SCI).

**Results:**

Sprague-Dawley rats underwent a contusive SCI at T9 using the MASCIS impactor. The rats developed hyperalgesia between days 21 and 42 post-SCI. Thermal hyperalgesia (TH) was determined by a decrease in hindpaw thermal withdrawal latency time (WLT) between days 21 and 42 post-SCI. Rats with TH were then treated with either vehicle (saline containing 0.25% NaOH) or NKCC1 inhibitor bumetanide (BU, 30 mg/kg, i.p.) in vehicle. TH was then re-measured at 1 h post-injection. Administration of BU significantly increased the mean WLT in rats (p < 0.05). The group administered with the vehicle alone showed no anti-hyperalgesic effects. Moreover, an increase in NKCC1 protein expression occurred in the lesion epicenter of the spinal cord during day 2–14 post-SCI and peaked on day 14 post-SCI (p < 0.05). Concurrently, a down-regulation of KCC2 protein was detected during day 2–14 post-SCI. The rats with TH exhibited a sustained loss of KCC2 protein during post-SCI days 21–42. No significant changes of these proteins were detected in the rostral region of the spinal cord.

**Conclusion:**

Taken together, expression of NKCC1 and KCC2 proteins was differentially altered following SCI. The anti-hyperalgesic effect of NKCC1 inhibition suggests that normal or elevated NKCC1 function and loss of KCC2 function play a role in the development and maintenance of SCI-induced neuropathic pain.

## Background

Spinal cord injury (SCI) and subsequent neuropathic pain can result in devastating motor and sensory deficits. Chronic neuropathic pain frequently develops following SCI and affects up to 70% of SCI patients clinically [1]. Effective analgesic therapy has been hampered by the lack of knowledge about the mechanisms underlying post-SCI neuropathic pain.

The GABAergic system plays an important role in spinal nociceptive processing. GABA receptors are found on pre- and post-synaptic sites of primary afferent terminals, as well as interneurons in laminae I-IV in the spinal cord dorsal horn [2]. GABAergic interneurons in the dorsal horn are important for nociceptive attenuation [3,4]. Subarachnoid implantation of GABA-producing neuronal cells in rats attenuates allodynia and hyperalgesia following excitotoxic injury [5]. Furthermore, administration of the GABA_A _receptor agonist muscimol prevents long-lasting potentiation of hyperalgesia following peripheral nerve injury [6]. However, the mechanism underlying the derangement of the GABAergic system during neuropathic pain state is unknown.

Normal GABAergic function is critically dependent on cation-chloride cotransporter activity, specifically inwardly directed Na^+^-K^+^-Cl^- ^cotransporter 1 (NKCC1) and outwardly directed K^+^-Cl^- ^cotransporter 2 (KCC2) [7-10]. Both NKCC1 and KCC2 are expressed in spinal cords and function to regulate intracellular Cl^- ^concentration. Increasing evidence suggests that changes of the transporter expression play a role in inflammatory or neuropathic pain [3,4,11,12]. Elevation of intracellular Cl^- ^can lead to GABAergic hypersensitivity by reversing both the Cl^- ^equilibrium potential (E_Cl_) and the normal inhibitory action of GABA. However, it remains unknown whether NKCC1 and KCC2 play a role in chronic hyperalgesia following SCI.

In the current study, a contusive SCI at T9 was induced in adult male rats using the MASCIS impactor. Inhibition of NKCC1 with its potent antagonist bumetanide (BU) had an anti-hyperalgesic effect in rats with chronic neuropathic pain following SCI. Moreover, transient increase in NKCC1 protein and down-regulation of KCC2 expression were detected in the spinal cord following SCI. The results imply that these Cl^- ^transporter proteins may be a potential target for the development of analgesics following SCI.

## Results

### Anti-hyperalgesic effects of bumetanide

**Figure 1:**
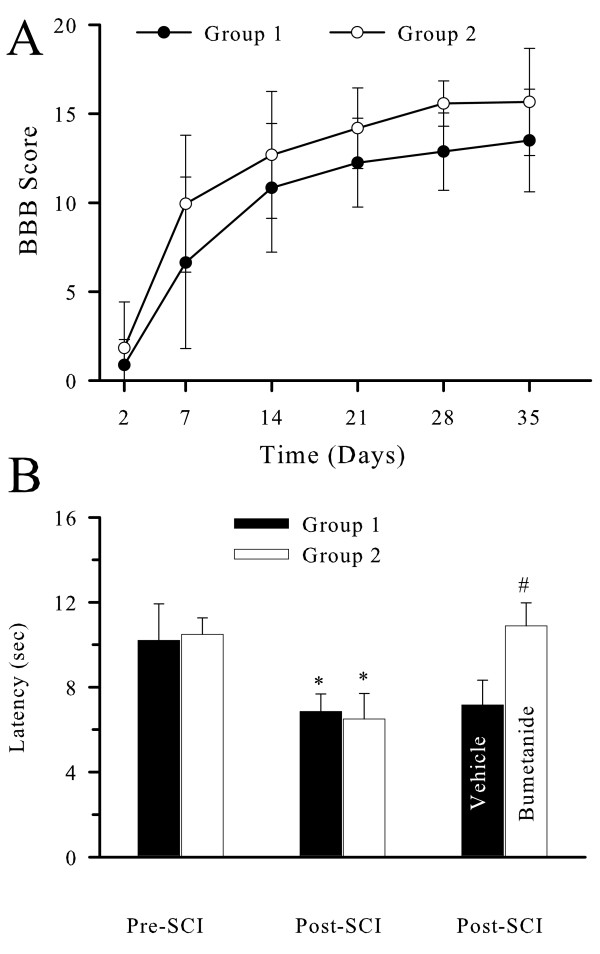
**Anti-hyperalgesic effects of bumetanide**. A. No difference in locomotor function following SCI in vehicle control and drug treatment groups. Animals were randomly divided into one of two groups (group 1 and 2). Locomotor function was monitored and Basso, Beattie, and Bresnahan (BBB) scores recorded on post-SCI days 2–42 in these groups. BBB scores recovered with time, and reached 13.5–15.7 by day 42 post-SCI. There were no significant differences in BBB scores between group 1 and group 2. B. Effects of Bumetanide on TH. TH was measured and the mean withdrawal latency time (WLT) recorded. Group 1 received vehicle (0.25% NaOH in saline, i.p.) as controls (n = 4) and group 2 received Bumetanide (BU 30 mg/kg, i.p., n = 8). After 1 h of treatment, TH was re-measured and the mean WLT recorded. Data are mean ± SD. * p < 0.05 vs. pre-SCI. # p < 0.05 vs. vehicle.

In order to assess the role of ion transporters in SCI-mediated hyperalgesia, it is important to verify that all animals experienced a similar degree of injury and exhibited similar locomotor function recovery prior to anti-hyperalgesic tests. Therefore, animals were randomly divided into one of two groups. In both group 1 and group 2, BBB scores showed classic locomotor function impairment after SCI (Figure 1A). BBB scores recovered with time, and reached 13.5–15.7 by day 42 post-SCI. There were no significant differences in BBB scores between group 1 and group 2 (Figure 1A).

In the experiments to test anti-hyperalgesic effects of bumetanide, group 1 served as the vehicle control and group 2 was treated with bumetanide. Mean withdrawal latency time (WLT) was 10.5 ± 1.7 s prior to SCI. In vehicle-treated animals (group 1), the mean WLT on 21–42 days post-SCI was reduced to 6.9 ± 0.8 s (p < 0.05, Figure 1B), which is consistent with our previous findings with SCI-induced TH [13]. Subsequent injection of the vehicle alone had no effects on the level of SCI-induced TH (7.2 ± 1.2 s). In contrast, following the administration of BU (30 mg/kg, i.p, group 2), there was a significant increase in mean WLT (from 6.5 ± 1.2 to 10.9 ± 1.1 s, p < 0.001), demonstrating an anti-hyperalgesic effect of BU. Moreover, to evaluate for potential non-specific analgesic drug effects, BU and vehicle controls were also administered in rats which did not demonstrate TH. No significant changes in mean WLT were observed in these animals (9.2 ± 1.8 s in vehicle-treated vs. 11.0 ± 0.74 s in BU-treated rats, p > 0.05). A lower dosage of BU (15 mg/kg, i.p.) gave rise to a similar anti-hyperalgesic effect as was observed with the higher dosage (WLT of 10.9 ± 2.8 vs. 7.2 ± 1.2 s in vehicle-treated rats, p < 0.05, n = 4).

### Expression of NKCC1 and KCC2 following SCI

**Figure 2:**
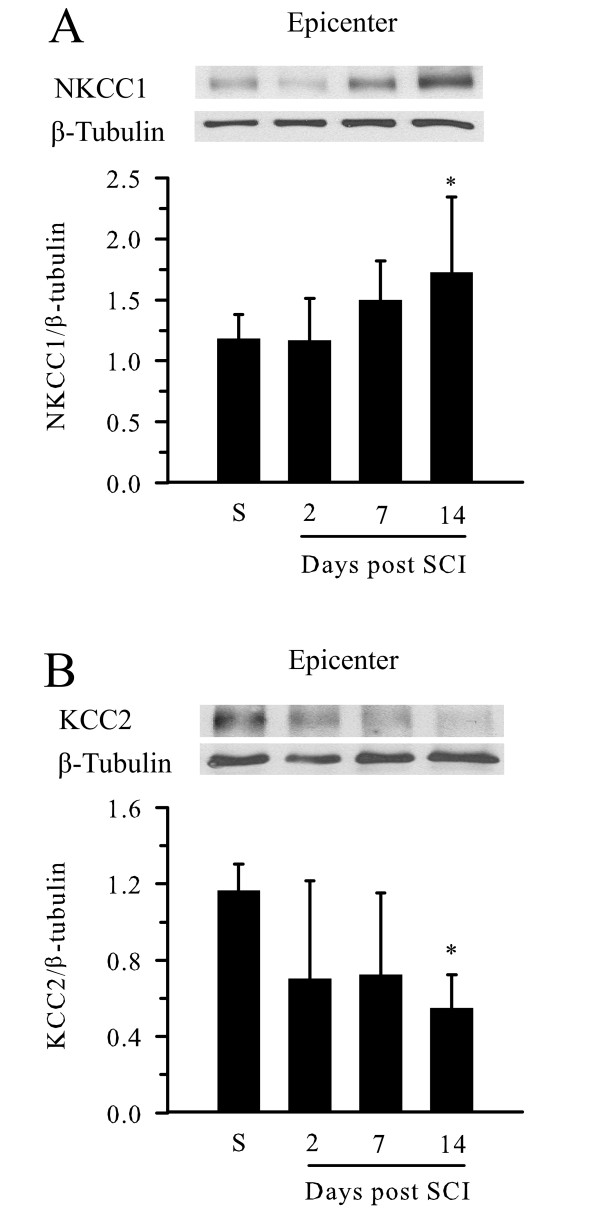
**Early changes in expression of NKCC1 and KCC2 proteins in epicenter spinal cord tissues following SCI**. A. NKCC1 protein expression in epicenter spinal cord tissues at 2, 7, and 14 days post-SCI. Sham (S) samples were acquired from animals subject to laminectomies without subsequent spinal cord contusion. Top panel, the blot was probed with anti-NKCC1 antibody (1:4000) and anti-βIII-tubulin antibody (1:5000). Lower panel, Densitometric analysis of the ratio of NKCC1/βIII-tubulin band intensity. Data were mean ± SD, n = 6. * p < 0.05 vs. Sham. B. Decrease in expression of KCC2 following SCI. Top panel, the blots were probed with anti-KCC2 antibody (1:500) and anti-βIII-tubulin monoclonal antibody (1:5000). Lower panel, summary of densitometry data for the ratio of KCC2/βIII-tubulin. Data were mean ± SD, n = 3. * p < 0.05 vs. S.

We investigated changes in NKCC1 protein levels in the epicenter of spinal cord tissue harvested at 2, 7, and 14 days post-SCI. A low level of NKCC1 protein was expressed in the spinal cord tissue at baseline (Figure 2A). βIII-tubulin was probed on the same blot to serve as control. Densitometric analysis of the NKCC1/βIII-tubulin band ratio intensities is shown in Figure 2A. NKCC1 was elevated on day 7 post-SCI and increased by ~60% on day 14 post-SCI (p < 0.05). In contrast, KCC2 protein was decreased on 2 and 7 days post-SCI and significantly fell by ~40% on day 14 post-SCI (p < 0.05, Figure 2B). However, no significant changes of βIII-tubulin were detected in the same sample. This data indicate a significant increase in the expression of NKCC1 and, conversely, a decrease in KCC2 expression at the epicenter on day 14 post-SCI (prior to the chronic phase of post-SCI neuropathic hyperalgesia).

**Figure 3:**
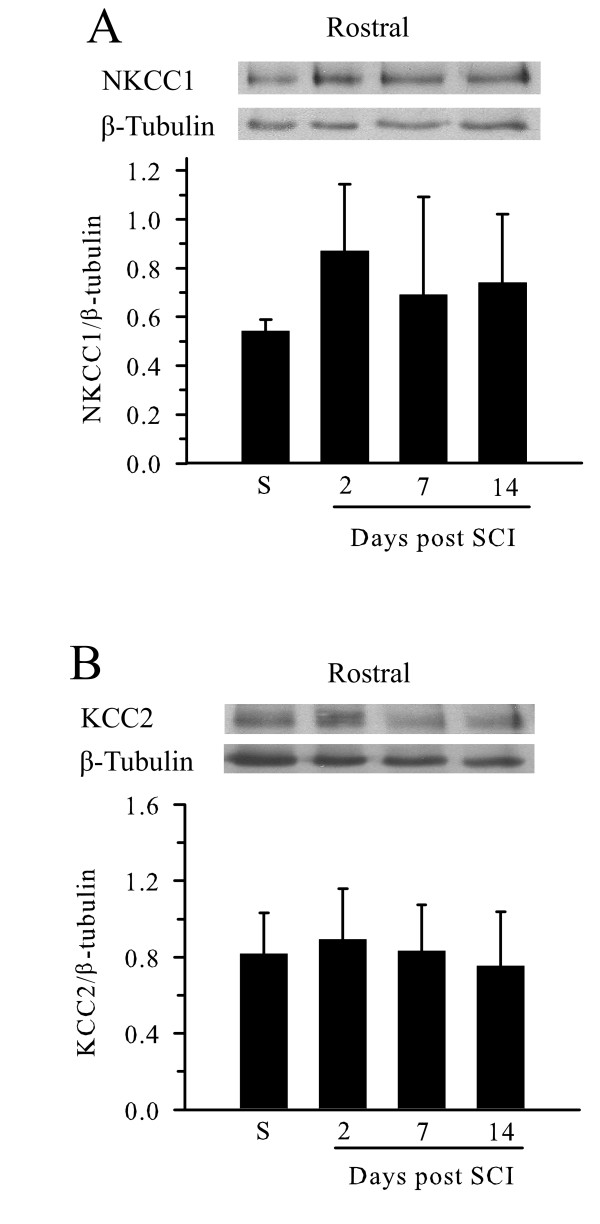
**Expression of NKCC1 and KCC2 in rostral spinal cord tissues after SCI**. A. Samples were prepared from spinal cord tissue rostral to the injury epicenter at 2, 7, and 14 days after SCI. Top panel, the blot was probed with anti-NKCC1 antibody (1:4000) and anti-βIII-tubulin antibody (1:5000). Lower panel, summary of densitometry data. Data are mean ± SD, n = 4. B. KCC2 expression post-SCI. Top panel, blots were probed with anti-KCC2 antibody (1:500) and anti-βIII-tubulin antibody. Lower panel, summary of densitometry data. Data are mean ± SD, n = 6.

To further establish that changes in NKCC1 and KCC2 are specific to the lesion epicenter of the spinal cord in response to SCI, we also examined spinal cord tissue rostral to the SCI injury epicenter. As shown in Figure 3A, NKCC1 protein level was low in sham control animals and tended to increase on days 2–14 post-SCI, but did not reach statistical significance. There were also no significant changes in KCC2 protein expression in rostral spinal cords on day 2–14 post-SCI (Figure 3B).

### Changes of NKCC1 and KCC2 in hyperalgesia rats following SCI

**Figure 4:**
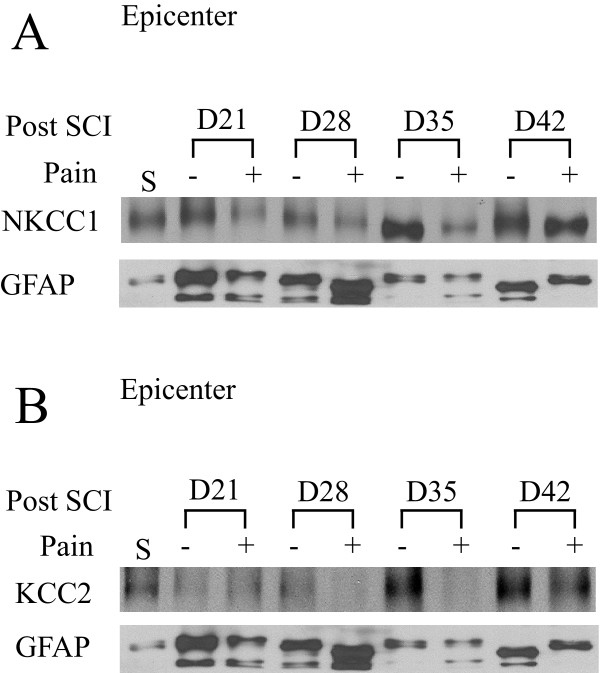
**Changes in NKCC1 and KCC2 expression in non-hyperalgesic and hyperalgesic rats**. NKCC1 protein expression in the injury epicenter spinal cord tissues at 21, 28, 35, and 42 days post-SCI. Sham (S) samples were acquired from animals subject to laminectomies without subsequent spinal cord contusion. Top panel, the blot was probed with anti-NKCC1 antibody and anti-GFAP (glial fibrillary acidic protein) antibody. Lower panel, the blot was probed with anti-KCC2 antibody and anti-GFAP antibody. Hyperalgesic rats (labeled as pain) were identified with the thermal hyperalgesia withdrawal latency test as described above. **(+): **hyperalgesia; (-): without hyperalgesia. n = 2.

We examined changes in NKCC1 and KCC2 protein levels in the contusion epicenter of spinal cord during days 21–42 post-SCI, when it was possible to determine the presence of chronic TH. As shown in Figure 4, a moderate reduction of NKCC1 occurred on days 21–35 post-SCI. KCC2 proteins remained down-regulated during days 21 and 35 post-SCI. Interestingly, in rats exhibiting no TH, both NKCC1 and KCC2 proteins remained unchanged as compared to sham. In contrast, TH rats displayed a sustained loss of KCC2 protein, with expression of KCC2 observed to be 34% and 40% of sham in lesion epicenter tissue at days 28 and 35 post-SCI, respectively. Injured spinal cord tissues develop neurodegeneration and severe glial scar formation after post-SCI day 20 [14]. Therefore, due to neuronal death and astrogliosis, no routine loading control markers (such as β-tubulin III for neurons and GFAP for glia) are appropriate for a quantitative immunoblotting analysis. In Figure 4, various changes of GFAP were illustrated, reflecting astrogliosis. As a result of this obstacle, no statistical analysis was performed. However, at least in the post-SCI day 28 samples, when higher levels of GFAP were detected in the spinal cord tissues in animals with neuropathic pain, the loss of KCC2 protein remained, which strongly suggests that the decrease in KCC2 expression is unlikely due to sample loading errors (insufficient protein loading).

## Discussion

### NKCC1 and KCC2 in hyperalgesia

The inhibitory action of GABA is a critical component of numerous neuronal circuits. It has been proposed that large, myelinated Aβ-fibers could antagonize nociceptive primary afferent inputs to the dorsal horn through inhibitory mechanisms mediated by interneurons. These interneurons release GABA which activates GABA_A _receptors on primary afferent terminals and produces primary afferent depolarization (PAD). PAD shunts the magnitude of incoming action potentials and decreases excitatory amino release at the primary afferent central terminals [3,4]. However, under inflamed conditions, PAD may be enhanced such that it leads to excessive depolarization of Aδ- and C-fibers above their thresholds for action potential generation.

Any injury-induced modification of this inhibitory action has the potential to alter the processing of nociceptive information in the spinal dorsal horn. Therefore, the generation of Cl^-^-dependent GABA_A _receptor response is critically dependent on the activity of NKCC1 and KCC2. NKCC1 knockout mice (NKCC1^-/-^) lack the GABA_A _receptor-mediated anion outward flux current [11]. NKCC1^-/- ^mice have deficits in thermal nociceptive threshoulds and display a decrease in Aβ-fiber-mediated touch-evoked allodynia following capsaicin injection [11]. Moreover, the NKCC1 antagonist bumetanide inhibits itch and flare responses to histamine in human skin, and attenuates phase I and II behavioral responses in the formalin model of tissue injury-induced pain [15]. After intra-colonic capsaicin injection in mice, NKCC1 plasma membrane expression and phosphorylation are increased in the dorsal spinal cord, although it is unknown whether it is accompanied with KCC2 down-regulation [16]. Inhibition of NKCC1 and TRPV1 attenuates capsaicin-induced allodynia [17]. These results suggest that NKCC1 plays a role in the initiation of hyperalgesia. On the other hand, intraplantar formalin stimulation triggers a significant decrease in KCC2 protein expression without changes in NKCC1 in the rat spinal cord [18]. Taken together, these studies suggest that alteration of Cl^- ^homeostasis by changes in NKCC1 and/or KCC2 may contribute to hyperalgesia development.

### NKCC1 and KCC2 in neuropathic pain

The current study examined the role of NKCC1 and KCC2 in chronic neuropathic pain following a contusion SCI. Immunoblotting showed a significant transient up-regulation of NKCC1 protein in the lesion epicenter of the rat spinal cord on day 14 post-SCI. This was accompanied by a concurrent down-regulation of KCC2 protein. Moreover, inhibition of NKCC1 with its potent antagonist BU significantly reduced pain behavior in rats. These results imply that altered NKCC1 and KCC2 expression may lead to GABAergic derangement and contribute to the induction and maintenance of the chronic neuropathic pain following SCI. BU inhibits NKCC1, but also KCC and other anion transport processes (e.g., Cl^-^/HCO3^- ^exchange, Cl^- ^channels etc.) at higher concentrations. However, the concentration of bumetanide (5 or 10 μM) is relatively specific for NKCC1 [19].

Partial nerve injury (induced by a sciatic cuff) disrupted anion homeostasis in lamina I neurons and shifted the normally inhibitory synaptic currents to excitatory, thereby increasing lamina I neuronal excitability *in vitro *[20]. The nerve injury was associated with a decrease in KCC2 mRNA and protein levels. Moreover, inhibition of KCC2 activity *in vivo *reduces mechanical and thermal nociceptive thresholds in control, and uninjured animals [20]. In addition, *in vivo *axonal injury causes a reduction of KCC2 mRNA in motor neurons and results in an increased intracellular Cl^- ^concentration and, consequently, GABA_A _receptor-mediated excitatory responses [21]. A recent report shows that this transient down-regulation of KCC2 [22] and the early pain behavior depends on activation of TrkB receptor via BDNF in nerve injury [12].

Post-translational modulation of KCC2 appears to be involved in the down-regulation of KCC2 protein. Exposing hippocampal neurons to H_2_O_2 _leads to a rapid dephosphorylation of KCC2 protein and a subsequent down-regulation of KCC2 protein [23]. A loss of tyrosine phosphorylation of KCC2 and a reduction of surface-expression of KCC2 in the plasma membrane is largely attributable to tyrosine phosphatases [23]. In our current study, reduction of KCC2 protein was detected in the epicenter region of the spinal cord on day 14–42 following SCI. It remains to be determined whether altered KCC2 phosphorylation is responsible for translocation between the plasma membrane and intracellular compartments in SCI. It is also unknown whether changes of KCC2 oligomerization play a role in neuropathic pain following SCI.

The significance of changes of NKCC1 and KCC2 proteins following SCI is not clear. Chloride homeostasis through the function of ionotropic GABA receptors has emerged as one important mechanism in the development of immature neurons [8]. NKCC1 phosphorylation stimulates neurite growth of injured adult sensory neurons [24]. Therefore, elevation of NKCC1 and down-regulation of KCC2 in lesion epicenter may reflect the cellular repair responses following SCI. However, the subsequent alteration of Cl^- ^homeostasis and GABAergic function causes neuropathic pain.

## Conclusion

In summary, we investigated the roles of two major intracellular Cl^- ^regulatory proteins, NKCC1 and KCC2, in chronic neuropathic pain following contusive SCI in the rat model. Contusive SCI caused a decrease in mean hindpaw thermal WLT in rats during the 21–42 day period post-SCI. Inhibition of NKCC1 with its potent antagonist BU significantly reduced pain behavior in rats. Immunoblotting showed a significant transient up-regulation of NKCC1 protein in epicenter of the rat spinal cord on day 14 post-SCI, which was accompanied by a concurrent down-regulation of KCC2 protein. Sustained reduction of KCC2 protein was prominent in epicenter of the spinal cord in TH rats. These results imply that altered NKCC1 and KCC2 expression may contribute to the induction and maintenance of the chronic neuropathic pain following SCI.

## Materials and methods

### Contusion SCI

All animal procedures used in this study were conducted in strict compliance with the National Institutes of Health *Guide for the Care and Use of Laboratory Animals *and approved by the University of Wisconsin Center for Health Sciences Research Animal Care Committee.

Adult male Sprague-Dawley rats (250–300 g) were anesthetized with gaseous isoflurane in oxygen (5% for induction, 3% for maintenance) throughout the duration of surgery. A T9 laminectomy was performed without disrupting the dura mater. Stabilization vertebral clamps were placed at T8 and T10 to the MASCIS Impactor (WM Keck Center for Collaborative Neuroscience, Rutgers University, Piscataway, NJ; Model II) and the spinal cord injured by releasing a 10 g rod (2.5 mm diameter) from a height of 12.5 mm as described previously [13]. Bupivacaine (Sensorcaine-MPF 0.25%, 0.20 ml) was administered subcutaneously as a local anesthetic. Throughout the procedure, body temperature was maintained at 37°C with a constant temperature heating pad. The animals were then returned to their cages after recovering from anesthesia. Animals underwent daily manual bladder expression until bladder control was re-established and each animal received Cefalexin antibiotic (0.10 mL; 330 mg/mL in saline) for 7 days post-injury. Six rats were designated as sham controls and underwent T9 laminectomy under anesthesia without the subsequent spinal cord contusion.

### Locomotor function

The functional neurological deficits due to the SCI were assessed by behavioral analysis using the Basso, Beattie, and Bresnahan (BBB) open-field locomotor test [25-27]. Animals were observed individually in an open field testing area consisting of a plastic wading pool. BBB scores were measured before injury (baseline) and on post-injury days 2, 7, 14, 21, 28, 35, and 42 from video recordings of 4 minutes per animal. BBB scores ranged from 0 (no hind limb movement) to 21 (normal movement – i.e. coordinated gait with parallel paw placement).

### Thermal withdrawal latency time

Thermal hyperalgesia (TH) was assessed using the hind paw withdrawal test to a thermal noxious stimulus [28]. TH is a sensitive and reproducible behavioral test of neuropathic pain, which is typically exhibited in the animals beginning approximately 21 days following SCI [13,29-31]. Rats were placed inside the Plexiglas apparatus (Plantar Test, Ugo Basile, Italy) and allowed to acclimatize. A noxious heat stimulus was applied to the plantar surface of the hind paw and the withdrawal latency time (WLT) recorded. Results of each test are expressed in seconds (s) as the mean of six withdrawal latencies (three from each hind paw). Animals were tested at different time points in order to avoid a training effect. Specifically, TH testing was performed prior to injury (baseline) and on post-injury days 21, 28, 35, and 42. A decrease in WLT of ≥ 2 sec from baseline is considered to be significant [31]. Onset of TH between days 21–42 post-injury occurred in nearly half of the injured animals, representing the chronic phase of post-SCI neuropathic hyperalgesia [13].

### Drug preparation

The NKCC1 antagonist bumetanide (BU) stock was prepared in 10 mg/mL of saline containing 0.25% NaOH. Control vehicle was prepared as 0.25% NaOH in saline. Control vehicle or BU (30 mg/kg) was randomly administered intraperitoneal (i.p.) into two groups (group 1, group 2) of rats exhibiting neuropathic hyperalgesia on post-SCI 21–42 days. TH was re-measured 1 h post-injection.

### Spinal cord harvesting

Spinal cords were harvested as two 7–10 mm sections: the epicenter of the contusion and rostral to the contusive lesion. The sections were immediately submerged in liquid nitrogen and stored at -80°C. Spinal cord samples were harvested from animals prior to onset of the chronic phase of neuropathic hyperalgesia (days 2, 7, and 14 following SCI) and on days 21, 28, 35, and 42 following SCI, when neuropathic hyperalgesia was developed. The spinal cords of sham control animals were harvested on day 2 post-surgery.

### Sample preparation and western blotting

The harvested spinal cord segments were homogenized in anti-phosphatase buffer (pH 7.4, mmol/L: 145 NaCl, 1.8 NaH_2_PO_4_, 8.6 Na_2_HPO_4_, 100 NaF, 10 Na_4_P_2_O_7_, 2 Na_3_VO_4_, 2 EDTA) and protease inhibitors as described previously [32]. The homogenate was centrifuged at 7000 rpm for 15 min at 4°C. The supernatant was retained and protein content of the supernatant was determined by the BCA protein assay (Pierce; Rockford, Ill). Protein samples (20 μg/lane) and pre-stained molecular mass markers (Bio-Rad; Hercules, CA) were denatured in SDS sample buffer. The samples were then electrophoretically separated on 6% SDS gels, and the resolved proteins were electrophoretically transferred to a PVDF membrane. The blots were incubated in 7.5% nonfat dry milk in Tris-buffered saline (TBS) overnight at 4°C, and then incubated for 1 h with a primary antibody. The blots were rinsed with TBS and incubated with horseradish peroxidase-conjugated secondary IgG for 1 h. Bound antibody was visualized using the enhanced chemiluminescence assay (ECL, Amersham Corp; Piscataway, NJ). Monocolonal antibody against NKCC1 (T4, 1:4000; Developmental Studies Hybridoma Bank; Iowa City, IA), and anti-KCC2 polyclonal antibody (1:500; Millipore, Billerica, MA) were used for detection of NKCC1 and KCC2, respectively. Anti-βIII-tubulin monoclonal antibody was used as a loading control (Promega; Madison, WI). Densitometric measurement of each protein band was performed with Un-Scan-It software (Silk Scientific; Orem, UT) and average pixel intensity was recorded.

KCC2 exists in monomeric and oligomeric structures [33]. In the current study, all protein samples were electrophoretically separated on 6% SDS gels in the presence of sulthydryl-reducing agent β-mercaptoethanol (5%). As reported by Blaesse et al [33], β-mercaptoethanol results in the disassembly of the three oligomeric structures of KCC2 oligomers. Therefore, we mainly analyzed changes of KCC2 monomers in the current study (~130 kDa).

### Statistical analysis

Comparisons between groups were made by student t-test and Mann-Whitney rank sum test where necessary (SigmaStat, Systat Software; Point Richmond, CA). A p < 0.05 was considered a statistically significant difference.

## Abbreviations

BBB: Basso, Beattie, and Bresnahan open-field locomoter test; Bu: Bumetanide; GFAP: glial fibrillary acidic protein; KCC2: K^+^-Cl^- ^cotransporter 2; NKCC1: Na^+^-K^+^-Cl^- ^cotransporter 1; PAD: primary afferent depolarization; SCI: spinal cord injury; TH: thermal hyperalgesia; WLT: withdrawal latency time.

## Competing interests

The authors declare that they have no competing interests.

## Authors' contributions

GM and SR were responsible for animal surgery, pain data collection, and analysis. SC was responsible for all immunoblotting experiments. GM, SR, DS, and DR were involved in experimental design. DS, CB, SC, and DR were involved in manuscript writing. All authors read and approved the final manuscript.
